# Amblyopia: An Uncommon Presentation of Paediatric Conversion Disorder

**DOI:** 10.1192/bjo.2022.360

**Published:** 2022-06-20

**Authors:** Chiro Islam Mallik, Mohammad Sayadul Islam Mullick

**Affiliations:** 1 Barnet, Enfield and Haringey Mental Health NHS Trust, Edgware, United Kingdom; 2 Bangabandhu Sheikh Mujib Medical University, Dhaka, Bangladesh

## Abstract

**Aims:**

Conversion disorder is common among children and adolescents, particularly in non-Western societies like in Bangladesh. Diverse presentation of the disorder makes it difficult to diagnose which ultimately may lead to poor prognosis.

**Methods:**

Patient X is a 10-year-old girl, a 5th grade student, hailing from a District town, Bangladesh, attending child and adolescent consultation services with the complaint of sudden loss of vision in both eyes for 3 weeks. Earlier, she underwent thorough examination and investigation and intervention by the different specialists (GP, ophthalmologist, and neurologist). At one point she was given zero power glasses. Her symptom was then fluctuating. She was not attending school as she was away for treatment purposes. X complained of not seeing lines of books when her parents tried to persuade her to do schoolwork at home. Eventually she was referred for psychiatric evaluation after no improvement. Assessment was completed and parental discord, violent act of father towards mother, overprotective as well as inconsistent parenting, attention seeking behaviour of X were identified. General and systemic examinations were normal. Her finding was inconsistent with any physical disorder. Investigation was normal. Thus, diagnosis of conversion disorder was given. General treatment (reassurance, psychoeducation, adopting daily life programme) along with specific treatment (symptom reduction by suggestion, relaxation, and family therapy) were provided. Improvement was noted during the follow-ups.

**Results:**

The conversion disorder of this girl happened at her period of transition from childhood to adolescence. Psychopathology of hysteria still is not clear despite huge research efforts. Based on psychodynamic explanations, the core psychopathologies of her symptoms are primary gain - resolution of her witnessing severe parental discord that she failed to cope with happened through her presenting symptoms without awareness though later she does in awareness; secondary gain- she receives additional reinforcement in the form of extra care, parental help seeking behaviour, unnecessary interventions, demand fulfilment that mostly present in awareness. Other psychopathologies are conversion- the patient's psychological distress converted to presenting somatic symptoms through an intrapsychic process; repression-la belle indifference of the patient has developed through a process of displacing distressful experience, from awareness that gives relief to the distress.

**Conclusion:**

Manifestations of conversion disorder can simply be explained as cry for help, give indication of the problem areas, help in understanding personality traits, making intervention and prevention plans. Early identification of stressors is crucial for the treatment of this disorder.

